# 

**Published:** 1835-07-01

**Authors:** 


### CONTENTS

OF THE

### MEDICAL QUARTERLY REVIEW,

### No. VIII. JULY 1, 1835.

---

Lectnres on the Diseases of the Urinary Organs. By SIR BENJAMIN C. BRODIE, Bart., V.P.R.S., Serjeant-Surgeon to the King, and Surgeon to St. George's Hospital . . . . . . 271

Clinical Illustrations of the more important Diseases of Bengal, with the Result of an Inquiry into their Pathology and Treatment. By WILLIAM TWINING, M.R.C.S. . . . . . . 290

Formulaire pour la Préparation et l'Emploi de plusieurs Nouveaux Médicaments; tels que la Morphine, la Codéine, l'Acide Prussique, la Strychnine, la Vératrine, l'Ether Hydrocyanique; le Sulfate de Quinine, la Cinchonine, l'Emétine, la Salicine, le Brôme, l'Iode, l'Iodure de Mercure, le Cyanure de Potassium, l'Huile de Croton Tiglium, les Sels d'Or, les Sels de Platine, le Chlore, les Chlornres de Chaux et de Soude, les Bi-carbonates Alcalins, la Grenadine, le Phosphore, l'Acide Lactique, l'Huile Volatile de Moutarde, &c. &c. Par F. MAGENDIE. Huitième édition, revue et augmentée

Formulary for the Preparation and Employment of several new Medicines; such as Morphia, Codeine, &c. By F. MAGENDIE . . 303

Outlines of Comparative Anatomy. By ROBERT E. GRANT, M.D., &c. Professor of Comparative Anatomy and Zoology in the University of London, &c. Part I. containing Osteology, Ligaments, and Muscles; illustrated with sixty-five Woodcuts . . . 316

A Practical Compendium of the Diseases of the Skin, with Cases. By JONATHAN GREEN, M.D. . . . . . 324

The Life of Thomas Linacre, Doctor in Medicine, Physician to King Henry VIII. By JOHN NOBLE JOHNSON, M.D. . . 337

Principles of the Treatment of Gout. By SIR CHARLES SCUDAMORE, M.D. 341

On Convulsions in Women during Pregnancy and Labour, and after Delivery. By A. VELPEAU . . . . 347

Asthma, its Species and Complications. Illustrated by Cases, and Plates coloured from Nature. By FRANCIS HOPKINS RAMADGE, M.D. 360

The Cyclopædia of Anatomy and Physiology. Edited by RT. B. TODD, M.D. 364

The Transactions of the Provincial Medical and Surgical Association 369

Human Physiology, by JOHN ELLIOTSON, M.D. Cantab., F.R.S., &c. 395

On the Deaths of some eminent Persons of Modern Times. By Sir HENRY HALFORD, Bart., M.D., G.C.H. . . . . . 411

A Dictionary of Practical Medicine. By JAMES COPLAND, M.D. F.R.S. &c. 416

Diseases of the Chest. By CHARLES J. B. WILLIAMS, M.D. . 419

A Grandmother's Advice to Young Mothers on the Physical Education of Children. By M. J., COUNTESS DOWAGER MOUNTCASHELL . 423

The Jamaica Physical Journal. Edited by JAMES PAUL, Esq. . 427

*Reviews continued.*

The Sphygmometer, &c. By Dr. E. S. Blundell . . 434

A Series of Twenty Plates, illustrating the Causes of Displacement in the various Fractures of the Extremities. By G. W. Hind, M.R.C.S. 437

A Therapeutic Arrangement and Syllabus of Materia Medica. By James Johnstone, M.D. . . . . . 440

Illustrations of the Botany, and other Branches of Natural History, of the Himalayan Mountains, and of the Flora of Cashmere. By J. Forbes Royle, Esq., F.L.S., &c. . . . . 441

Medical Zoology; being a faithful Representation and Description of the Animals which belong to the Materia Medica. By Dr. J. F. Brandt and Dr. J. T. C. Ratzeburg . . . . 445

### Original Communications.

Cases extracted from the Note-book of Henry Davies, M.D. . 446
Case of Morbid Adhesion of the Placenta. By Dr. Litchfield . 449
Case of Intus-susception, successfully treated. By John Howship, Esq. 451
Case of Traumatic Tetanus, occurring in the Norfolk and Norwich Hospital. Reported by Mr. Archibald Dalrymple, Norwich . 454
Account of several Cases of Carditis: with Remarks. Communicated to the Harveian Society, by William Stroud, M.D. . . 459

### COLLECTANEA.

### PATHOLOGY AND PRACTICE.

Report of the Surgical and Ophthalmic Institution of the University of Berlin, for the Year 1833 . . . . 474
An Opium-Eater . . . . . 482
Cases of Paralysis of Individual Nerves of the Face. By Dr. Christison 483
Accidental Occlusion of the Vagina, forming an Obstacle to Delivery. By C. Hoillemin, D.M.P. . . . . 488
Dr. Hope on the Sounds of the Heart . . . 489
On the Reduction of Strangulated Hernia . . . 495
History of a Unique Case of Heart Disease. By Samuel Hanna, A.M. 501
On the Tricuspid Valve, or Safety-Valve . . . 503
Fractured Pelvis: Hæmorrhage; Suppuration within the Pelvis . 505

### MISCELLANEOUS.

Imaginary New Editions . . . . 506
The Rapidity of Thought . . . . 508
Inquest at Dublin . . . . . 509
Diseases and Wounds of Plants . . . . 510

### INTELLIGENCE.

Society of Apothecaries. Regulations to be observed by Students intending to qualify themselves to practise as Apothecaries in England and Wales ib.

Biography of Dr. Maton . . . . . 519

Meteorological Register . . . . 521

Notice to Subscribers . . . . . 522

